# Pharmacological screening of silibinin for antischizophrenic activity along with its acute toxicity evaluation in experimental animals

**DOI:** 10.3389/fphar.2023.1111915

**Published:** 2023-02-02

**Authors:** Qurat Ul Ain, Uzma Saleem, Bashir Ahmad, Iqra Khalid

**Affiliations:** ^1^ Department of Pharmacology, Faculty of Pharmaceutical Sciences, Government College University, Faisalabad, Pakistan; ^2^ Hamza College of Pharmaceutical and Allied Health Sciences, Lahore, Pakistan

**Keywords:** silibinin, acute oral toxicity, schizophrenia, ketamine, oxidative stress

## Abstract

Silibinin (SIL), a flavolignan extracted from the medicinal plant “silybum marianum (milk thistle)”, has traditionally been used to treat liver disease. This phytochemical has displayed neuroprotective properties, its activity against schizophrenia is not elucidated. The present study was designed to evaluate the antipsychotic potential of silibinin and probe its toxic potential. The acute oral toxicity study was assessed as per OECD 425 guidelines. Animals were divided into two groups of female rats (n = 6): one group served as the normal control and the other group received a 2,000 mg/kg dose of SIL. We also evaluated the antipsychotic potential of SIL. To this end, animals were divided into six groups (n = 10) of mice for both the preventive and curative protocols. Group I (CMC 1 mL/kg) served as the normal control and received CMC 1 mL/kg; group II was the diseased group treated with ketamine (10 mg/kg) i.p; group III was the standard group treated with clozapine 1 mg/kg; groups IV, V, and VI served as the treatment groups, receiving SIL 50, 100, and 200 mg/kg, respectively, orally for both protocols. Improvement in positive symptoms of the disease was evaluated by stereotypy and hyperlocomotion, while negative symptoms (behavioral despair) were determined by a forced swim test and a tail suspension test in the mice models. The results suggested that the LD_50_ of SIL was greater than 2,000 mg/kg. Moreover, SIL prevented and reversed ketamine-induced increase in stereotypy (*p* < 0.001) and behavioral despair in the forced swim and tail suspension tests (*p* < 0.001). Taken together, the findings suggest that silibinin is a safe drug with low toxicity which demonstrates significant antipsychotic activity against the positive and negative symptoms of schizophrenia.

## 1 Introduction

Phytochemicals derived from plant, animals, and marine sources have played a vital role in alleviating major human suffering. They have demonstrated therapeutic potential in reducing the risk of major health problems such as cancers, cardiovascular diseases, and different central nervous system (CNS) disorders (Kumar and Khanum, 2012). Phytochemicals have gained attention due to their therapeutic value and the fact that they have fewer adverse effects. Most people seem to believe that natural products are free of any kind of adverse properties, Paracelsus, known as the father of toxicology, stated that “all substances are poisons; it is the right dose that differentiates remedy and poison.” Hence, assessing the toxic potential of natural products is of vital importance (Newman and Cragg, 2012).

Schizophrenia is a severely debilitating disease that affects patients’ thoughts, behavior, perception, and memory (Faludi et al., 2011). The disease affects 1%–1.5% of the global population (Mcgrath et al., 2008) and is rated seventh among the most costly diseases (Ross and Margolis, 2005). Symptoms of schizophrenia cluster into three groups: positive symptoms include hallucination, thought disorder, and delusion; negative symptoms include lack of interest and pleasure and low social interaction; cognitive symptoms include memory and thinking problems (Tandon et al., 2013). The etiology of this disease is still unclear, although the dopamine hypothesis remains its mainstay. After 1980, the glutamate hypothesis was proposed as the establishing basis of schizophrenia (Yadav et al., 2019).

Pharmacotherapeutic agents for schizophrenia were introduced in 1950s and have been active for positive symptoms but ineffective against negative symptoms and cognitive deficits (Purdon et al., 2001). Typical antipsychotics have been dopamine (D2) receptor antagonists such as haloperidol and prochlorperazine. Second generation drugs (clozapine and quetiapine) were then approved for negative symptoms and cognitive impairments (Wang et al., 2019). These antipsychotic drugs are the mainstay of treatment but are unsuccessful at counteracting the symptoms and progression of the disease. Some serious adverse effects as extrapyramidal symptoms, dyslipidemia, diabetes, and hypertension are related to antipsychotic drug use (Brown, 2012; Fell et al., 2012). Therefore, there is a need to investigate new and more effective therapeutic alternatives with fewer adverse effects to increase adherence and therapeutic outcomes.

Silibinin (SIL) is a flavolignan derived from silymarin, a compound that is extracted from the seeds and fruit of the milk thistle herb (*Silybum marianum*). This plant belongs to the Asteraceae family and its fruit has been famous for use in medicines for centuries. Silybin A and Silybin B are two stereoisomers of silibinin (Mashhadi Akbar Boojar et al., 2020). Flavonoids are known for their antioxidant antiviral, anti-inflammatory, and neuroprotective effects (Hassan et al., 2022). According to previous findings, SIL acts as an antioxidant, anti-inflammatory, anticarcinogenic, and growth promoting agent and is used as a hepatoprotective agent—especially for liver cirrhosis (Chu et al., 2004; Shanmugam et al., 2008; Yin et al., 2011). Moreover, it is also useful for dermatological conditions and skin aging and can act as an antiviral and protective agent against retinal diseases (Singh and Agarwal, 2009; Ahmed-Belkacem et al., 2010). Furthermore, SIL is a neuroprotective agent against neural damage, ethanol-induced brain damage, Aβ-induced memory impairment, and neurotoxicity associated with lipopolysaccharide. Its antidepressant activity is also established (Mazzio et al., 1998; Yan et al., 2015), but its antipsychotic property has not been investigated. Acute toxicity study is basic and preliminary to evaluating the general safety and toxicity of a substance (Baig et al., 2022); however, despite its wide usage, toxicity data is not yet available for SIL (Kennedy et al., 1986).

Hence, the present study aims to evaluate the acute oral toxicity of SIL and determine its antipsychotic potential.

## 2 Materials and methods

### 2.1 Drug and chemicals

This study used silibinin (CAS # 22888-70-6), ketamine (CAS # 1867-66-9), clozapine (CAS # 5786-21-0), sodium hydroxide (CAS # 1310-73-2), sodium carboxymethyl cellulose, isoflurane (CAS # 26675-46-7), pyrogallol (CAS # 87-66-1), DTNB (5, 5-dithiobis-(2-nitrobenzoic acid) (CAS # 2516-96-3), Folin-Ciocalteu (CAS # F9252), potassium phosphate monobasic, and sodium phosphate monobasic. All chemicals were of analytical grade and were purchased from Sigma Aldrich (China).

### 2.2 Animals

Adult Wister female rats weighing 200–300 g were used to examine acute toxicity. Male and female Swiss albino mice (approximately 30 g) were purchased from Government College University Faisalabad, Pakistan, and were used for behavioral studies of schizophrenia. The animals were kept in the department’s animal house at 22 ± 2ᵒC with a relative humidity of 44%–56% and a 12 h light–dark cycle. They were provided with a standard rodent pellet diet and water. The animals were acclimatized to handling and the room and apparatus before experimentation commenced. Ethical approval for the animal studies was obtained from the Animal Ethics Committee of Government College University Faisalabad, reference number GCUF/ERC/2015.

### 2.3 Acute oral toxicity studies

The OECD’s (Organization for Economic Co-operation and Development) 425 Guidelines were followed for performing an acute oral toxicity study. In this study, two groups of healthy adult Wister rats were used (n = 6). The animals were fasted overnight but were allowed access to water. Group I received 0.5% carboxy methyl cellulose (CMC) (1 mL/100 g) and was designated as normal control. Group II, the treatment group, received 2000 mg/kg of SIL orally. Initially, only one animal received SIL (2000 mg/kg) by a single oral dose and observations were made for 24 h. If the animal survived, four further animals were administered single oral doses of SIL (2,000 mg/kg). The animals were observed for mortality, changes in general behavior, weight change, and any kind of allergies for 14 days (Choi et al., 2021).

### 2.4 Hematological and biochemical analysis

After 14 days, animals were anesthetized with 3%–5% isoflurane diluted with oxygen. Blood samples were collected after anesthesia by cardiac puncture, and hematological and biochemical parameters were evaluated. Hematological parameters included WBC, RBC, Hb, platelet count, hematocrit (HCT) value, mean corpuscular hemoglobin concentration (MCHC), mean corpuscular volume (MCV), and mean corpuscular hemoglobin (MCH) using a hematology analyzer (Norma, USA). For biochemical tests, plasma and serum were separated and biochemical parameters were estimated. The lipid profiling of cholesterol and triglycerides was measured using their specific kits (CC1132, and CC1302 by MTD Diagnostic, Italy, respectively). Liver function tests for alanine transaminase (ALT) and aspartate transaminase (AST) were performed using their appropriate kits (CC1223, CC1213 by MTD Diagnostic, Italy, respectively) (Steinberg et al., 2019).

### 2.5 Determination of oxidative stress markers

#### 2.5.1 Preparation of organ tissue homogenates

After euthanasia with 3%–5% isoflurane anesthesia diluted with oxygen, the mice brains were collected from all animals in each group with scarification; the brains were washed in a cold solution of normal saline. Brain tissue homogenates were prepared by adding a 0.1 M phosphate buffer of pH 7.4 in a 1:10 ratio. Homogenates were then centrifuged at 6,000 rpm at 4^ᵒ^C for a period of 10 min. Supernatants were then separated for performing different biochemical tests. Organs such as the heart, kidney, lung, liver, and spleen were also collected and weighed separately. Tissue homogenates were prepared similarly to brain homogenate.

#### 2.5.2 Estimation of glutathione level

In 1 mL of tissue homogenate of each of the above organs (heart, brain, kidney, liver, and spleen), 10% trichloroacetic acid (1 mL) was added for precipitation of protein. In 4 mL of phosphate solution, supernatant and 5,5-dithiobis-2-nitrobenzoic acid (DTNB) reagent (0.5 mL) were added and absorbance was measured at 412 nm. The level of GSH is expressed in μg or level of glutathione per mg of protein. GSH level was then measured using the formula (Bhangale and Acharya, 2016):

GSH level=Y−0.00314÷0.0314×DF÷Bt×Vu

where *Y* is the absorbance at 412 nm, *Bt* is homogenate of brain tissue, *DF* is dilution factor, and *Vu* is the aliquot volume (1 mL).

#### 2.5.3 Estimation of the catalase level

Mixed into 0.05 mL of supernatant (tissue homogenate of each organ—heart, brain, kidney, liver, and spleen) was 1.95 mL of phosphate buffer (50mM, pH 7.0). The above solution was mixed with 1 mL of 30 mM hydrogen peroxide (H_2_O_2_) and absorbance was recorded at 240 nm. Values were expressed in micromoles of H_2_O_2_ oxidized/min/mg of protein. The catalase level was determined by formula (Anwar et al., 2021).

CAT activity=O.D÷E×vol. of sample×mg of protein

where *O.D* is the change in absorbance/minute while *E* is the extinction coefficient (0.071 mmol^4^/cm) of H_2_O_2_.

#### 2.5.4 Estimation of superoxide dismutase level

Tissue homogenates (0.1 mL) of heart, brain, kidney, liver, and spleen were added to 2.8 mL of 0.1 M potassium phosphate buffer (pH 7.4) and 0.1 mL pyrogallol solution. The absorbance of the mixture was measured on a UV spectrophotometer at 325 nm (X. Li, 2012).

By using a regression line equation, SOD level was thus determined:

Y=0.0095 x+0.1939

#### 2.5.5 Determination of malnodialdehyde level

The supernatant of 1 mL of each organ (heart, brain, kidney, liver, and spleen) and 1 mL of thiobarbituric acid (4.0 mM) were mixed in 100 mL of glacial acetic acid. A mixture of 3 mL of sample was shaken and set aside for 15 min and kept for cooling. Centrifugation was done at 35,000 rpm for 10 min. Absorbance was taken at 532 nm. Malnodialdehyde was quantified as micromole per mg of protein. The following formula was used for MDA quantification:

$$Conc.\;of\;MDA=Abs532\times 100\times Vt÷\left(1.56\times 10^{5}\right)\times wt\times Vu$$

where *Abs*_532_ is absorbance, *Vt* is mixture volume (4 mL), 1.56 × 10^5^ is the molar extinction coefficient, *wt* is the weight of brain, and *Vu* is the volume of aliquot (1 mL) (Bhangale and Acharya, 2016).

#### 2.5.6 Estimation of nitrite level

Griess reagent was used to determine nitrite level by spectrophotometer. A mixture was made by adding equal quantities of Griess reagent and tissue homogenate of each organ (heart, brain, kidney, liver, and spleen). Incubation was done for 10 min and then absorbance was taken at 546 nm. Nitrite level was calculated by the following regression line equation for sodium nitrite (Anwar et al., 2021):

Y=0.003432x+0.0366

#### 2.5.7 Estimation of protein content

Three solutions were prepared. A) 1% NaK tartrate in H_2_O; B) 0.5% CuSO_4_.5H_2_O; C) 2% Na_2_CO_3_ dissolved in 0.1 N NaOH in water. Reagent 1 was formed by mixing Solutions A (48 mL), B (1 mL), and C (1 mL). Reagent 2 was prepared in 2:1 part (one part folin-phenol [2 N]: one part H_2_O). For protein content determination, we added 0.2 mL tissue homogenate of each organ (heart, brain, kidney, liver, and spleen) to 4.5 mL of reagent and then incubated it for 10 min. Reagent 2 (0.5 mL) was then added to the mixture and incubated again for 30 min. The absorbance of the mixture was noted at 660 nm. The protein contents were determined by the following regression line equation of BSA and expressed in μg/mL (Lowry et al., 1951):

Y=0.00007571x+0.0000476

### 2.6 Evaluation of antipsychotic potential

#### 2.6.1 Study protocol

The effect of SIL on ketamine-induced schizophrenia-like behavioral alterations was evaluated by a procedure following Ben-Azu et al. (2018) with modifications for both the preventive and treatment protocols. Animals were grouped in two phases, with six groups for the preventive study and six groups for the curative tests (n = 10).

**Table:** 

Group I	Vehicle control group (0.5% CMC 1 mL/kg i.p)
Group II	Negative control group (ketamine 10 mg/kg i.p)
Group III	Positive control group (clozapine 1 mg/kg i.p)
Group IV	SIL 50 mg/kg
Group V	SIL 100 mg/kg
Group VI	SIL 200 mg/kg orally

Ketamine was given to all groups except group I1; group III received clozapine; groups IV, V, and VI received SIL with a 30 min interval after ketamine. In the preventive protocol, SIL was administered 30 min before ketamine in groups IV, V, and VI while, in the curative protocol, SIL was given to these groups after 30 min of ketamine administration.

### 2.7 Behavioral tests

#### 2.7.1 Ketamine-induced stereotypy

The effect of SIL on stereotypy induced by ketamine was assessed by any repetitive, functionless activity of mice in a transparent observation cage (23 × 20 × 20 cm). Stereotypical behavior was counted as head movement, intermittent sniffing, licking, and chewing for 2 min after 5, 10, 15, 30, and 60-min intervals. After each observation, the chamber was cleaned with 70% ethanol to remove any odor.

Initially, animals were individually placed in cylindrical metal cages for 15 min prior to the test to acclimatize to the environment. After ketamine administration, the mice were immediately placed in a cage at the base. Scoring for stereotypy was set as:

0 = no stereotypy,

1 = head movements,

2 = intermittent sniffing,

3 = chewing,

4 = licking intensely (Eneni et al., 2020).

#### 2.7.2 Ketamine-induced hyperlocomotion

Locomotor activity was measured by an activity meter. An acclimatization time of 10 min was given to each animal before testing. After acclimatization, animals were individually placed in an actophotometer, and total locomotor activity was counted for 5 min. Before and after ketamine administration, animals were placed in the actophotometer. Observations were made in a square closed arena of 30 cm that was equipped with infrared-light-sensitive photocells. The total number of crossings was automatically calculated for 5 min. After each test session, the chamber was cleaned with 70% ethanol (Bishnoi et al., 2008).

#### 2.7.3 Ketamine-induced immobility

##### 2.7.3.1 Immobility in forced swim test

The mice were acclimatized in a glass cylinder of 33 cm height and 20.5 cm diameter that contained water 20 cm deep at 25ᵒC. They were removed after 15 min and returned to the cage. This was referred to as “training day”. The next day, the mice were again placed in a cylinder for 6 min. Their swimming activity was recorded after the initial first 2-min period. Their duration of immobility was determined where “immobility” refers to a condition when no other activity was present despite that needed to keep the animal`s head above the water. The animals were then dried with a towel after the test was completed (Chen et al., 2021).

##### 2.7.3.2 Immobility in tail suspension test

To determine immobility, a tail suspension test was performed. The mice were suspended from a tail hanger with adhesive tape that was wrapped around their tails from about 2 to 3 cm from the tip, and about 30 cm above the floor in a box (25 × 25×30 cm). The animals were observed for 6 min and the duration of their immobility was measured manually. Immobility was determined when the animal hung passively and remained motionless (Arruda et al., 2008).

### 2.8 Statistical analysis

All results were presented as mean ± SEM. Statistical significance difference was analyzed by one-way ANOVA followed by Dunnett`s *t*-test and two-way ANOVA followed by Bonferroin`s multiple comparison test. *p < 0.05, **p < 0.01, and ***p < 0.001 denoted mild, moderate, and high levels of significance.

## 3 Results

### 3.1 Acute toxicity studies

#### 3.1.1 Effect of treatments on body and organ weight

The body weights of the treatment groups were measured daily until the 14th day. Significant increases in body weight were seen in group (SIL 2,000 mg/kg) treated animals compared with Day 1 (Table 1). There was no significant difference in the weights of the selected organs compared with the normal control group (Table 2).

**TABLE 1:** Estimation of mice body weights during acute oral toxicity study.

Groups		Body weights g)	
Treatments	Day 1	Day 7	Day 14
Normal control	228.5 ± 3.86	233.83 ± 3.80	238.50 ± 3.86
SIL (2,000 mg/kg)	239.3 ± 5.50	253.50 ± 6.92*	264.50 ± 7.28**

Values expressed as mean ± SEM; n = 6, where *p < 0.05, **p < 0.01 compared to Day 1.

**TABLE 2:** Estimation of organ weight in treatment groups during acute oral toxicity study.

Organs	Weights g)	
	Normal control	SIL (2000 mg/kg)
Liver	3.31 ± 0.38	3.30 ± 0.12
Heart	0.31 ± 0.13	0.33 ± 0.21
Lung	0.74 ± 0.11	0.80 ± 0.45
Spleen	0.28 ± 0.13	0.31 ± 0.08
Kidney	0.82 ± 0.27	0.83 ± 0.31

Data presented as mean ± SEM, n = 6.

#### 3.1.2 Treatment effect on hematology parameters

On the 14th day, blood was collected from each group through cardiac puncture. Table 3 showed that, except for the platelet and WBCs count, all parameters show a non-significant difference compared to the control group. The platelet count significantly decreased and the WBCs count was significantly increased compared to the control group.

**TABLE 3:** Estimation of hematological parameters in acute oral toxicity.

Parameters	Control	SIL (2000 mg/kg)
Hb (g/dL)	12.33 ± 0.42	14.25 ± 0.44
Total RBC’s (x10^6^/ml)	05.93 ± 0.15	06.26 ± 0.09
HCT (%)	31.53 ± 0.84	34.88 ± 1.04
MCV (fL)	54.81 ± 0.35	56.51 ± 0.46
MCH (pg)	20.04 ± 0.88	21.69 ± 0.96
MCHC (g/dL)	37.50 ± 0.92	40.00 ± 1.21
W.B.C (X10^3^/ml)	03.63 ± 0.06	09.60 ± 0.18*
Platelet count (X10^3^/ml)	819.16 ± 1.95	547.3 ± 6.85^

Data represented as mean ± SEM; n = 6, *p < 0.05 significant increase, ^*p* < 0.05 significant decrease vs. control group.

#### 3.1.3 Effect of treatments on biochemical markers in the acute oral toxicity study

The SIL (2,000 mg/kg) treated group showed a significant increase in all biochemical markers (renal, hepatic, and lipid profile) compared with the normal control group. Uric acid and protein levels had parallel values as compared to control (Table 4).

**TABLE 4:** Estimation of biochemical markers in acute oral toxicity.

Biochemical markers	Normal control	SIL (2000 mg/kg)
Cholesterol (mg/dL)	57.93 ± 1.20	79.23 ± 1.63***
Triglycerides (mg/dL)	56.1 ± 1.43	81.08 ± 1.54***
ALT (U/L)	39.33 ± 1.60	55.17 ± 2.28***
AST (U/L)	63.33 ± 1.56	77.5 ± 1.76***
Uric acid (mg/dL)	05.2 ± 1.62	3.45 ± 1.23
Protein (g/dL)	07.12 ± 2.13	7.93 ± 2.02
Creatinine (mg/dL)	1.16 ± 1.23	2.13 ± 1.18*
Bilirubin (mg/dL)	1.5 ± 1.21	2.74 ± 1.13*

Data represented as mean ± SEM; n = 6, *p < 0.05 significant increase, ***p < 0.001 high significant increase vs. control group.

#### 3.1.4 Oxidative stress markers in acute toxicity studies

**FIGURE 1:**
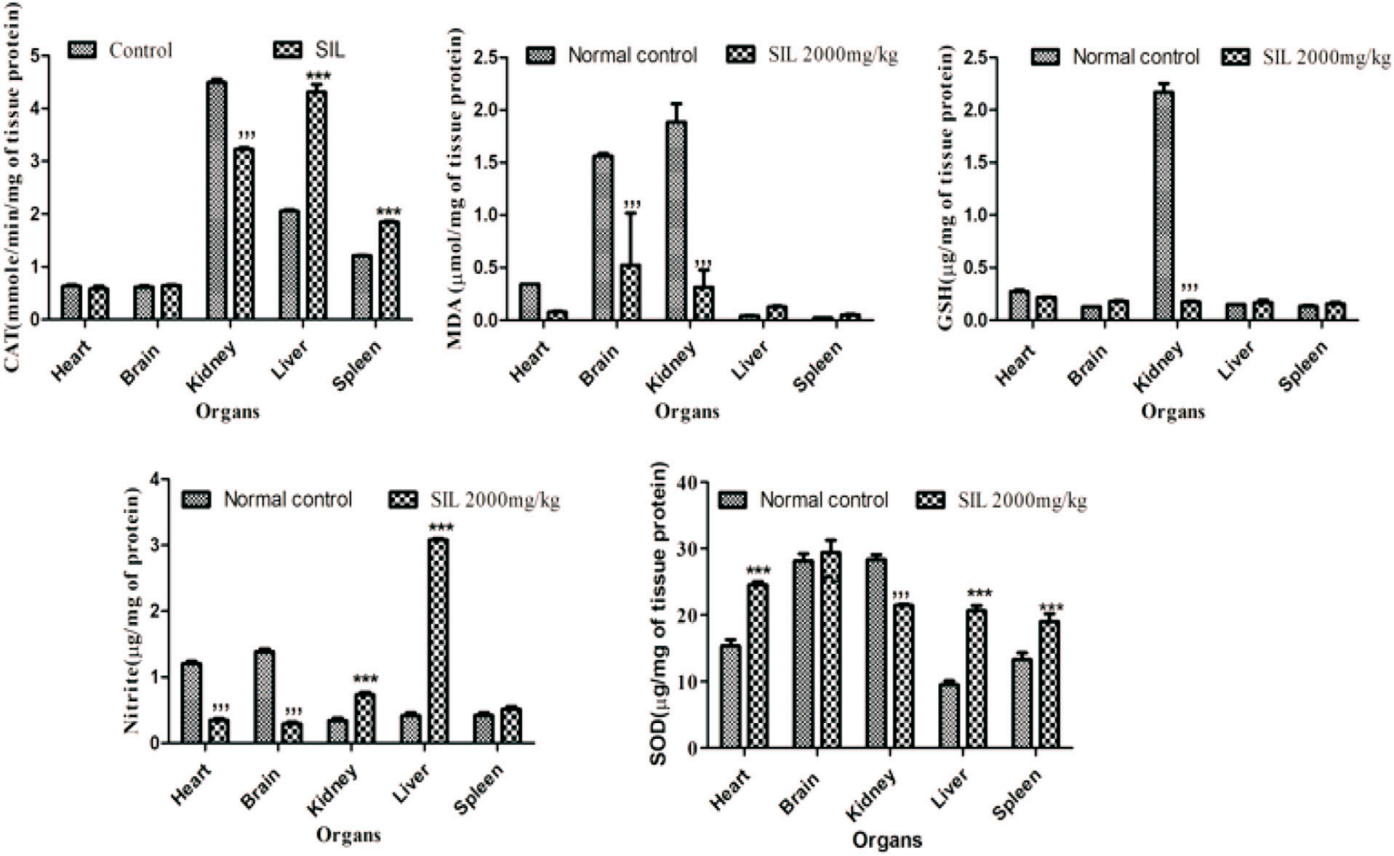
Effect of normal control and SIL treatment on oxidative stress markers. Data presented as mean ± SEM. n = 6. Here, ***p < 0.001 high significant increase vs. the control group, and ***p < 0.001 significant decrease compared with the normal control group.

Oxidative stress biomarkers were estimated in selected organs (heart, brain, kidney, liver, and spleen) to identify signs of toxicity and cellular destruction. A significant reduction in endogenous antioxidants (SOD, GSH, and CAT) was observed in the kidney while MDA levels in the kidney had risen when compared to the control group. The spleen and liver displayed a significant rise in SOD and CAT *versus* control. MDA and nitrite levels were significantly reduced in the brain and heart compared to normal control groups (Figure 1).

#### 3.1.5 Histopathological analysis

**FIGURE 2:**
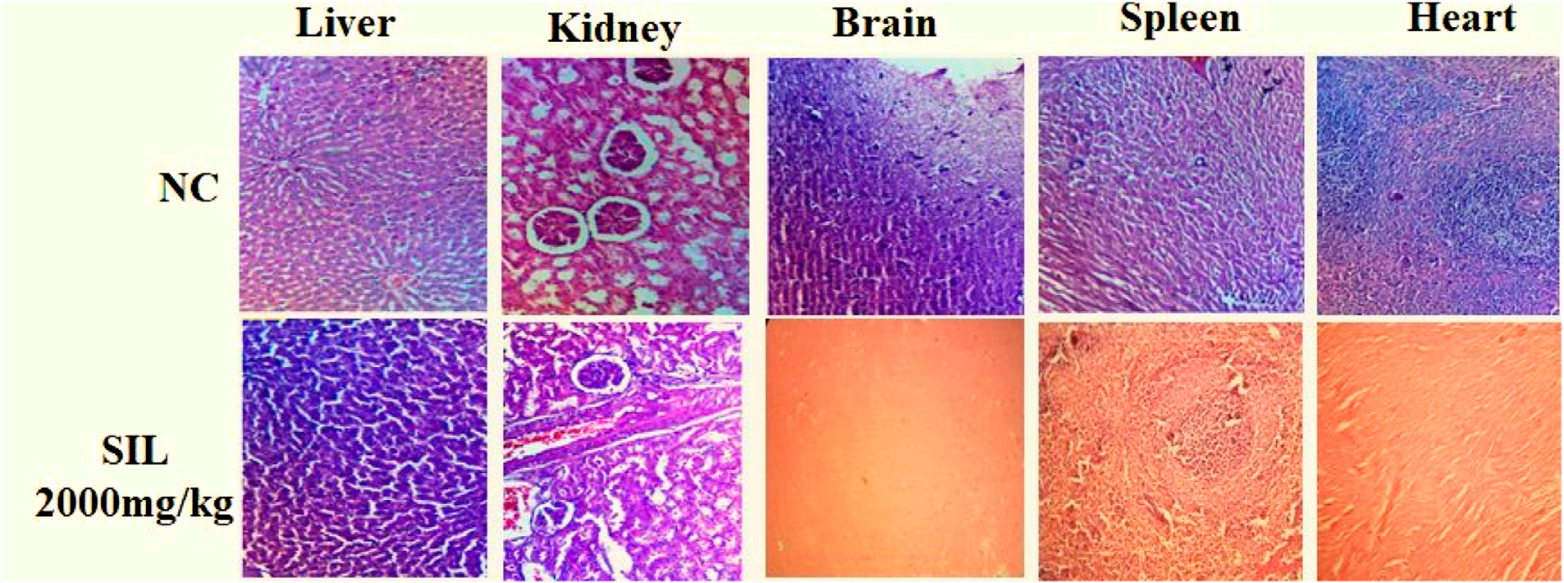
Histogram of selected organs in treated groups in acute toxicity study.

Histopathological analysis showed normal cellular architecture, and no significant change was observed in selected organs after treatment compared to the normal control group (Figure 2).

### 3.2 Behavioral observations

#### 3.2.1 Effect of SIL on ketamine-induced stereotypy in preventive and treatment protocols

**FIGURE 3:**
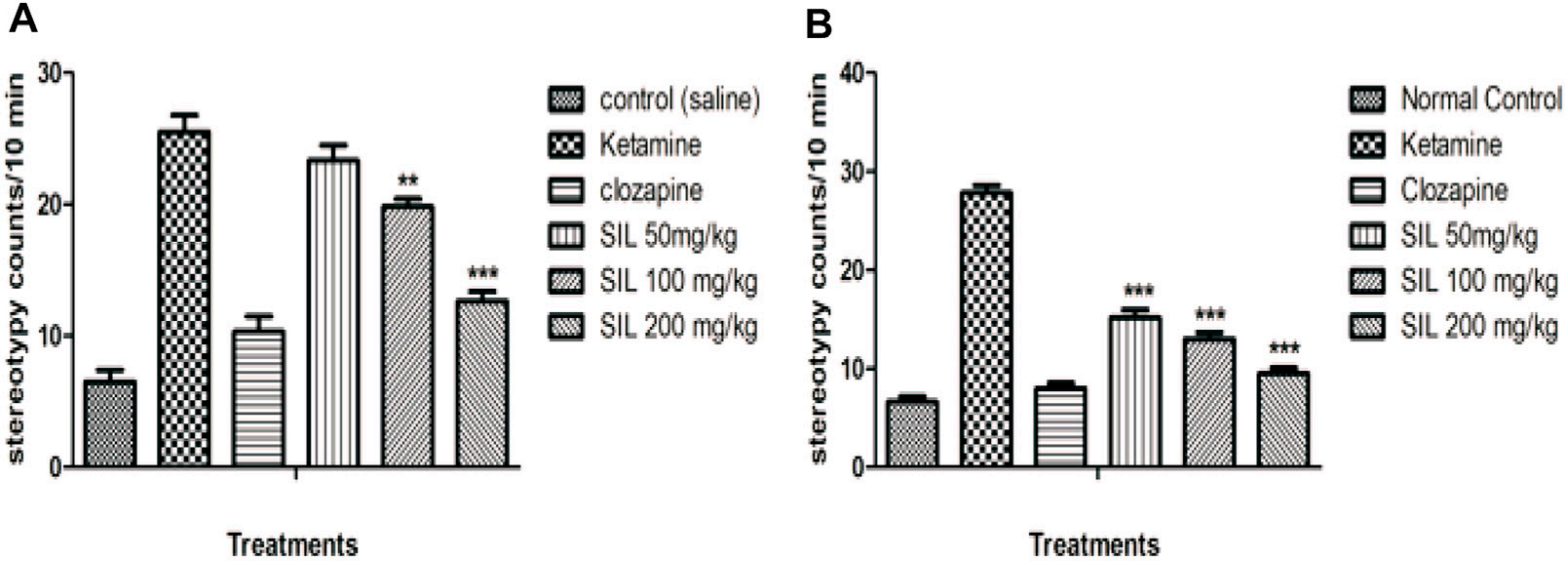
Effect of SIL on ketamine-induced stereotypy in preventive **(A)** and curative **(B)** protocols. Values expressed here as mean ± SEM; n = 10, where **p < 0.01, ***p < 0.001 compared to the ketamine group.

Stereotypy (in minutes) was counted in all treatment groups in both the preventive and treatment protocols. Animals treated with ketamine showed significant stereotypic behaviors compared to the control group. Observations were made between 10 and 30 min in both the treatment and preventive protocols. SIL at all dose levels reduced the stereotypy counts compared to the ketamine group in the treatment protocol, and hence, there was a highly significant (*p* < 0.001) and dose-dependent response to decreasing stereotypy (Figure 3B). In the preventive protocol, SIL at 100 mg/kg showed a significant (*p* < 0.01) reduction in stereotypy while SIL (200 mg/kg) and clozapine showed highly significant (*p* < 0.001) decreases in the stereotypy score (Figure 3A).

#### 3.2.2 Effect of SIL on ketamine-induced hyperlocomotion in preventive and treatment protocols

**FIGURE 4:**
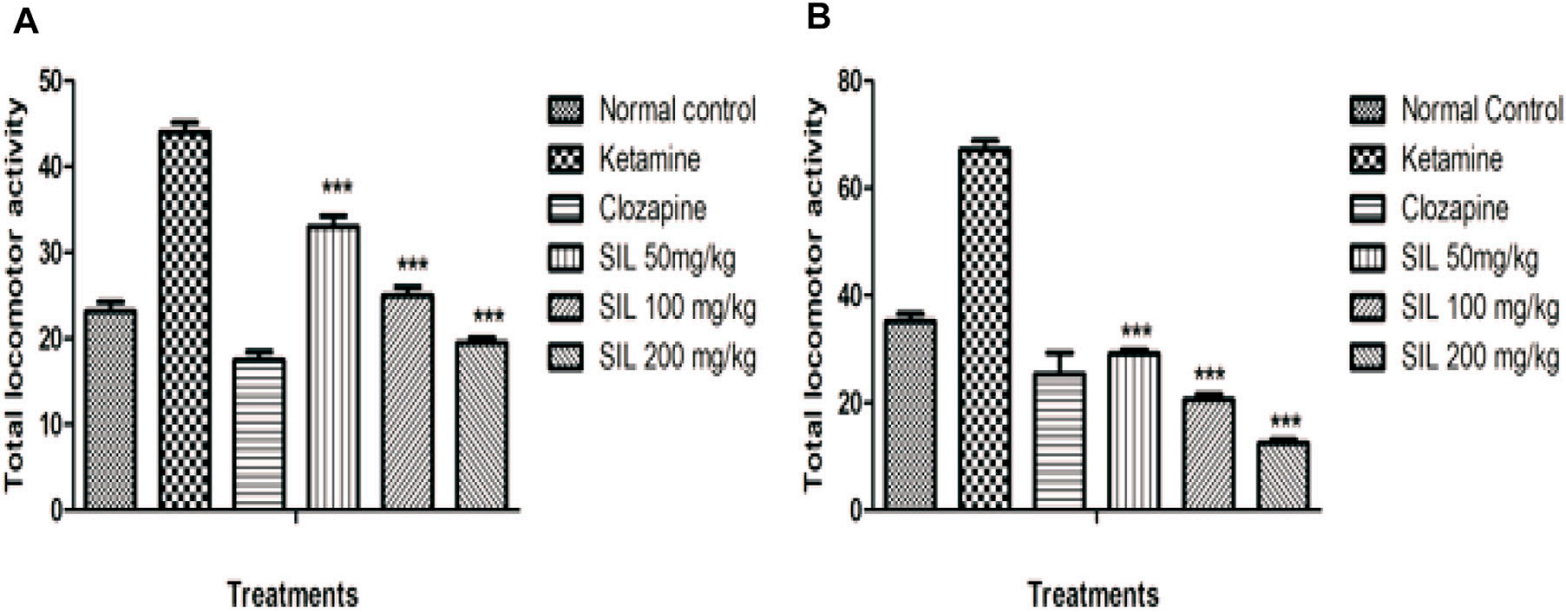
Effect of SIL on ketamine-induced hyperlocomotion in preventive **(A)** and curative **(B)** protocols. Values are expressed as mean ± SEM; n = 10, ***p < 0.001 as compared to the ketamine group.

In the open field test, locomotor activity was significantly increased in the ketamine group when compared with control in both the preventive and curative treatment protocols. While the results showed SIL at all doses and clozapine significantly (*p* < 0.001) reduced the hyperlocomotion induced by ketamine in both protocols. Moreover, in the preventive protocol, SIL at 100 mg/kg showed a significant reduction (*p* < 0.01) in the number of crossings. Hence SIL represented a dose-dependent decrease in hyperlocomotion in comparison to the ketamine group (Figures 4A, B).

#### 3.2.3 Effect of SIL on ketamine-induced immobility by forced swim test in preventive and treatment protocols

**FIGURE 5:**
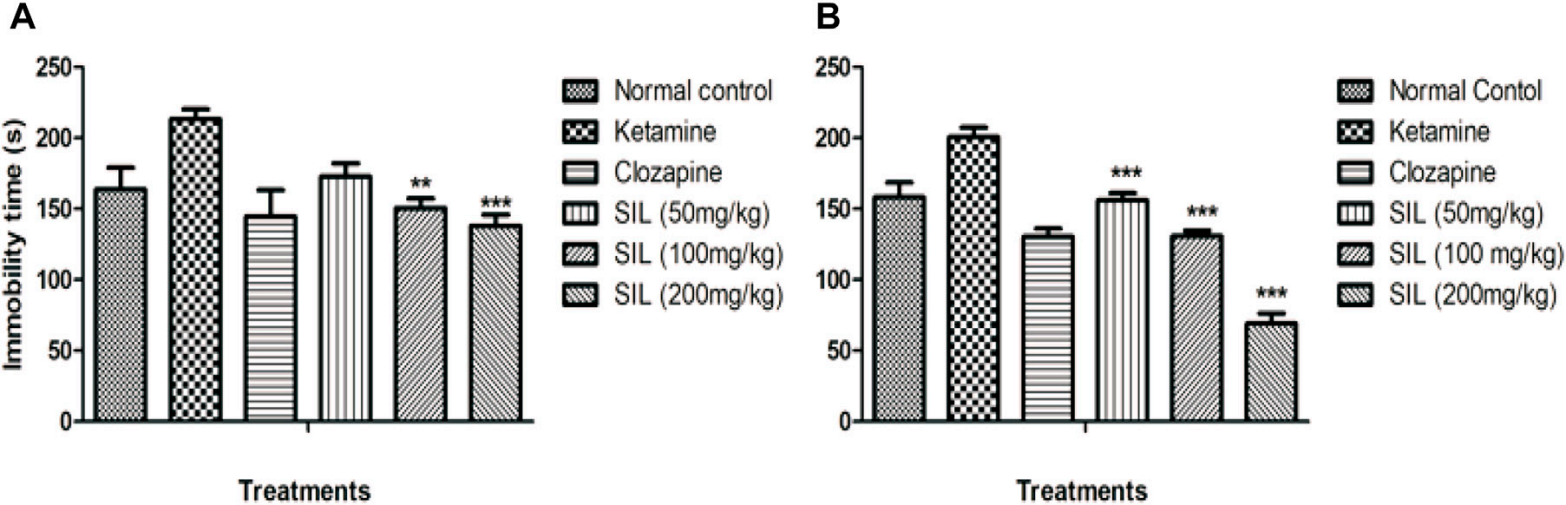
Effect of SIL on ketamine-induced immobility in forced swim test in preventive **(A)** and treatment **(B)** protocols. Values are represented here as mean ± SEM; n = 10, where **p < 0.01, ***p < 0.001 compared to the ketamine group.

Behavioral despair was evaluated by immobility time in the forced swim test in all treatment groups in the preventive as well as treatment protocols. Ketamine injection produced a marked increase in immobility time that connotes behavioral despair when compared with the saline group. SIL showed a significant (*p* < 0.001) and dose-dependent reduction in immobility in all treatment groups in the treatment protocol. In the preventive protocol, only the highest dose group of 200 mg/kg exhibited a significant reduction in immobility time (Figures 5A, B).

#### 3.2.4 Effect of SIL on ketamine-induced behavioral despair by tail suspension test in preventive and treatment protocols

**FIGURE 6:**
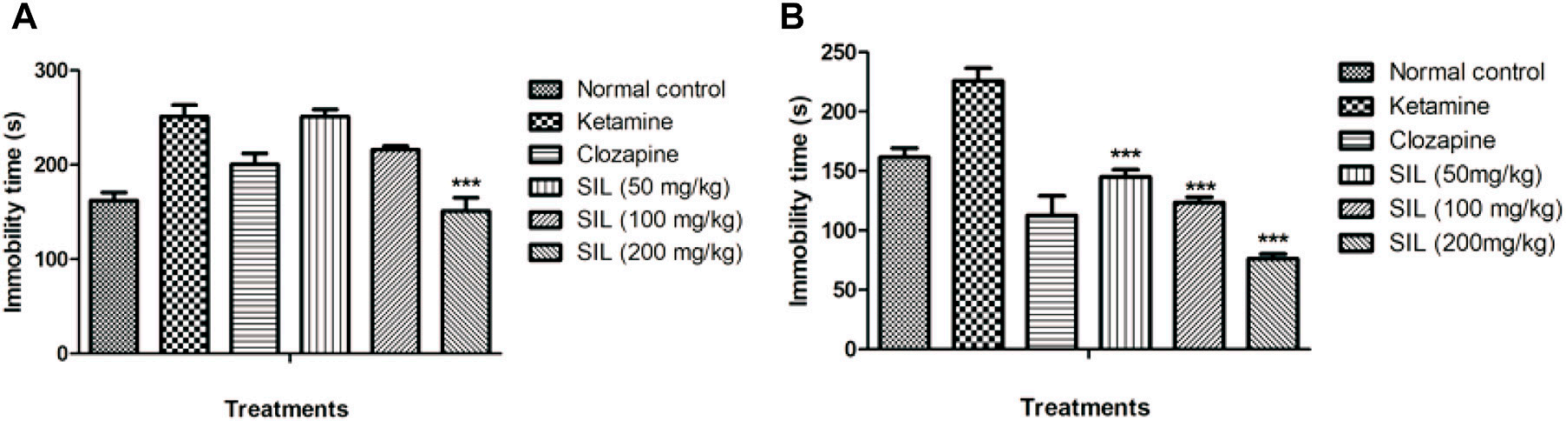
Effect of SIL on ketamine-induced immobility by tail suspension test in preventive **(A)** and treatment **(B)** protocols. Values expressed as mean ± SEM; n=10, ***p<0.001 compared to the ketamine group.

Behavioral despair was also evaluated by the tail suspension test in treatment and preventive protocols (Figures 6A, B). Ketamine (10 mg/kg) significantly induced immobility and behavioral despair compared with the control group. In the curative protocol, SIL significantly (*p* < 0.001) and dose dependently reduced immobility time in mice. Only clozapine and SIL 200 mg/kg showed a highly significant (*p* < 0.001) reduction in ketamine-induced immobility (Figures 6A, B).

## 4 Discussion

Phytochemicals are derived from natural origins and provide cost-effective, valuable, and relatively safe alternatives for allopathic medicines (Saleem et al., 2022; Datta et al., 2021). The WHO and FDA have emphasized the validation of safe and effective uses of natural chemicals by conducting scientific studies (Saleem et al., 2017) and preliminary toxicity studies are important for the safe use of phytochemicals. Silibinin is known for its various pharmacological properties, like treating diabetes, hypertension, dyslipidemia; it has been utilized in China, the USA, and Europe for thousands of years for its folkloric properties against liver diseases and its hepatoprotective properties (Mazzio et al., 1998; Takke and Shende, 2019). However, comprehensive knowledge about its safety and toxicity is still lacking. Hence, this study was designed to evaluate the LD_50_ of SIL by following OECD guidelines in an animal model which will help to determine the safe dose range for managing its adverse effects in clinical practice (Anwar et al., 2021). The main purpose of this study was to observe animals after a single high-dose administration for any life-threatening toxicities or adverse effects. The results of this study provide evidence that SIL, according to the globally harmonized classification system, can be placed in the fifth group where LD_50_ > 2000 mg/kg in a lower toxicity class (Saleem et al., 2017). Undeniably, hematological and biochemical estimations remain the hallmarks of the health status of the body, as these are sensitive to changes produced by chemical toxins. Platelets are known to play an important role in coagulation (Li et al., 2008); results have revealed a decrease in the level of platelet counts, which might be linked to the hemostatic property of SIL. Moreover, the elevated WBC count is suggestive of the immunopotentiation property of SIL by endogenous defense system to the adverse effects of silibinin. As the liver and kidney are primary organs for the metabolism and elimination of chemicals, alterations in the liver’s function parameters and renal function tests are known to be connected to the toxicity of said organs (Moses et al., 2018). Moreover, an increase in cholesterol and triglyceride showed the hyperlipidemic effect of SIL, while elevation in ALT and AST might show some hepatic injury. Liver damage may cause increased permeability of cell membranes that could result in a release of aminotransferases into the blood (Ogunlana et al., 2013). These findings also demonstrate that elevated renal function parameters may also be related to mild renal injury. Moreover, histopathological findings appear quite normal, and no significant change appeared in the selected organs studied.

Continuous exposure to reactive oxygen species in the living system causes oxidative stress damage, adversely affecting proteins, lipids, nucleic acid, and whole-body systems. Necrosis, apoptosis, and irreversible cellular damage are linked with oxidative stress (Jones and Libert, 2006; Nair et al., 2015). The current studies estimated the endogenous levels of oxidants and antioxidants in the major organs of animals’ bodies after treatment with SIL. There was a significant reduction in antioxidants (GSH, CAT, and SOD) in the kidney and an increase in the oxidant MDA. SIL as a lipophilic compound might be excreted through the kidney, indicative of a decrease in its antioxidant parameters. Moreover, an increase in the level of antioxidants (SOD and CAT) in the liver and spleen and a reduction in MDA and nitrite in the brain and heart shows the increased antioxidant property of SIL in these respective organs.

Another aim of the present study is to demonstrate the efficacy of SIL in protecting and reversing ketamine-induced psychosis-like behavioral manifestations. In recent research on schizophrenia, the use of animal models that can present different symptoms of schizophrenia (positive, negative, and cognitive) has become more popular (Chatterjee et al., 2012b). Schizophrenia, being a complex neuropsychiatric disease, involves different neurochemical alterations, including the effect of dopamine, gamma amino butyric acid (GABA), and glutamate on motor neuron function (Vasconcelos et al., 2015). Our results support previous findings that ketamine (an NMDA blocker) can induce multifarious behavioral alterations (i.e., stereotypy, hyperlocomotion, and behavioral despair) that are relevant to the positive and the negative symptoms of the disease. Ketamine-induced stereotypy and hyperlocomotion have remained important measures of positive symptoms (Ben-Azu et al., 2016). These symptoms have been previously linked with the NMDA receptor blockade that is present in inhibitory GABA neurons in the mesolimbic region of the brain. This inhibition causes behavioral alterations called stereotypy that are characterized by aimless and repetitive motor activity (Chatterjee et al., 2012b). Furthermore, ketamine may act as an indirect dopamine agonist, which might also explain its valuable role in behavioral stimulations (Irifune et al., 1991). Clozapine is a widely used drug for schizophrenia patients in clinical settings as well as a reference drug for researchers on schizophrenia (Vasconcelos et al., 2015). Thus, our study demonstrated the effect of SIL to prevent and reverse ketamine-induced stereotypy (as evidenced through decreased stereotypic head movements, intermittent sniffing, intense licking, and chewing in mice) and hyperlocomotions (number of crossings), revealing its antipsychotic potential against positive symptoms (Ben-Azu et al., 2018).

Furthermore, SIL was also evaluated for its effect on negative symptoms of schizophrenia by ketamine-induced immobility duration by a forced swim test and tail suspension test. Both models represented behavioral despair and a lack of motivational behavior suggestive of the negative symptoms of disease (Chatterjee et al., 2012a). Our findings are in line with previous research as intraperitoneal injection of a sub-anesthetic dose of ketamine demonstrates behavioral despair related to negative symptoms of schizophrenia represented by increased immobility in the forced swim and tail suspension tests (Eneni et al., 2020). The administration of SIL reduced the immobility period in these tests comparable with clozapine. This and other antipsychotic drugs act to manage the negative symptoms of schizophrenia, generally acting as antagonists on 5HT2 receptors (Glatt et al., 1995; George et al., 2020). Accordingly, our findings imply that SIL can act as antipsychotic drug and may attenuate the positive and negative symptoms of schizophrenia, warranting further investigation of its mechanism of action.

## Conclusion

From the findings of this acute toxicity study, it can be concluded that the LD_50_ of SIL is more than 2000 mg/kg, and there was no sign of morbidity and mortality seen in animals, except for some alterations in renal function test parameters and lipid profile. More importantly, this study revealed that SIL can attenuate positive and negative symptoms of schizophrenia and can act like an atypical antipsychotic. However, the results suggest that it must be evaluated in future chronic and repeated administration of silibinin to completely ensure its safety. Antipsychotic potential can be determined in more detail by long-term studies. Furthermore, a more scientific approach can further identify the mechanistic study of silibinin as an antipsychotic drug.

## Data Availability

The raw data supporting the conclusions of this article will be made available by the authors, without undue reservation.
